# Pinealectomy affects bone mineral density and structure - an experimental study in sheep

**DOI:** 10.1186/1471-2474-12-271

**Published:** 2011-11-24

**Authors:** Marcus Egermann, Christian Gerhardt, Alain Barth, Georges J Maestroni, Erich Schneider, Mauro Alini

**Affiliations:** 1 AO Research Institute, Davos, Switzerland; 2 Clinic for Orthopedic Surgery, University Hospital Heidelberg, Germany; 3 Department of Neurosurgery, University of Berne, Switzerland; 4 Center for Experimental Pathology, Locarno, Switzerlan

## Abstract

**Background:**

Osteoporosis and associated fractures are a major public health burden and there is great need for a large animal model. Melatonin, the hormone of the pineal gland, has been shown to influence bone metabolism. This study aims to evaluate whether absence of melatonin due to pinealectomy affects the bone mass, structure and remodeling in an ovine animal model.

**Methods:**

Female sheep were arranged into four groups: Control, surgically ovariectomized (Ovx), surgically pinealectomized (Px) and Ovx+Px. Before and 6 months after surgery, iliac crest biopsies were harvested and structural parameters were measured using μCT. Markers of bone formation and resorption were determined. To evaluate long term changes after pinealectomy, bone mineral density (BMD) was analyzed at the distal radius at 0, 3, 9, 18 and 30 months.

**Results:**

Cancellous bone volume (BV/TV) declined after 6 months by -13.3% Px and -21.5% OvxPx. The bone loss was due to increased trabecular separation as well as decreased thickness. The histomorphometric quantification and determination of collagen degradation products showed increased bone resorption following pinealectomy. Ovariectomy alone results in a transient bone loss at the distal radius followed by continuous increase to baseline levels. The bone resorption activity after pinealectomy causes a bone loss which was not transient, since a continuous decrease in BMD was observed until 30 months.

**Conclusions:**

The changes after pinealectomy in sheep are indicative of bone loss. Overall, these findings suggest that the pineal gland may influence bone metabolism and that pinealectomy can be used to induce bone loss in sheep.

## Background

Treatment of osteoporotic fractures remains generally an unsolved problem[1]. The application of conventional implants for fracture fixation in osteoporotic bone is limited mostly because of the weakened bone structure[2]. Prior to the implementation of new treatment options into the clinical situation, in-vivo testing of new treatments or devices is required to prove efficiency and safety. Hence, an animal model is indispensable for demonstrating the benefit of any new modality sufficiently different from the standard treatment. The FDA Guideline recommend that agents should be evaluated in two different animal species including ovariectomized rats and a second non-rodent large animal model which possesses Haversian systems and remodelling patterns similar to the human situation[3]. Therefore a large animal model of osteoporosis is required[4].

Contrary to humans, naturally occurring osteoporosis is hardly found in animals, and osteoporosis can only be induced in them. The ovariectomized rat is the animal model studied most for osteoporosis. But, biomechanical implant testing is limited and cortical bone remodelling is different than in humans[5,6]. A comparison of the other common experimental animals to potentially simulate human osteoporosis shows that the sheep fulfils many of the qualifications[7]. Ovariectomy (Ovx) alone has been demonstrated to induce a significant decrease of bone mass density in sheep[8,9] but the changes in the bone mass have been observed directly after Ovx only and no long-term effect was found [10]. The bone loss is much lower in comparison to human osteoporosis and would be better termed osteopenia[4].

Melatonin is the principal substance secreted by the pineal gland with roles of biologic regulation of circadian rhythms, aging and reproduction. There is considerable interest in the potential role of melatonin in bone metabolism[11]. The finding that bone marrow cells of mice are capable of synthesizing melatonin suggest that this hormone may have local regulatory actions in bone[12]. Consistent with the possibility of direct bone actions, melatonin stimulates cell proliferation and activity on osteoblasts[13] and enhances human adult mesenchymal stem cell differentiation into osteoblasts[14]. Daily injections of 50 mg/kg/day of melatonin significantly increased bone mineral density and trabecular structure in mice[15]. These skeletal effects probably were caused by the melatonin-mediated down-regulation of the RANKL-mediated osteoclast formation and activation. Furthermore, melatonin is suspected to be involved in the age-related disorder of osteoporosis[16]. Accordingly, the secretion of melatonin declines progressively with increasing age. The menopause is time-related with a substantial decrease in melatonin secretion and an associated increase in the rate of pineal calcification[17,18]. A recent report also shows that obese women (who have a low risk for osteoporosis) have a higher daytime secretion of melatonin compared with non-obese women and that the melatonin level in the obese women is associated with a reduction in serum bone turnover markers[19]. Ladizesky et al concluded from their results that melatonin modifies bone remodeling after ovariectomy and the effect may need adequate concentrations of estradiol[20,21]. Pinealectomy reduces the nocturnal expression of melatonin and causes an increase of collagen degradation products in rats[22]. Thus, there is circumstantial evidence that melatonin may alter bone metabolism.

The rationale of this study is to establish a large animal model of osteoporosis by decreasing the endogenous levels of melatonin using pinealectomy. Our approach is to investigate the short and long term effect of Px compared to Ovx and OvxPx on the bone mineral density, bone structure and bone metabolism in sheep.

## Methods

### Study design

The European laws on animal experimentation were observed strictly during the entire study and the animal research protocol was approved by the appropriate institutional animal care committee (Veterinary Department of the Canton of Grisons). Mature and identically bred female White Swiss Alpine Sheep (3.1 ± 0.5 yrs, weight: 67.7 ± 2.7 kg) were arranged into four groups: untreated control (C; n = 8), ovariectomy (Ovx; n = 6), pinealectomy (Px n = 6) and ovariectomy in combination with pinealectomy (OvxPx; n = 6). Before and 6 months after surgery, bicortical iliac crest biopsies were harvested to determine structural parameters using μCT and histomorphometry. Urine and serum samples were collected three times (day 28, 14 and 7) before surgery as baseline level and at 3, 6 and 9 months to analyze biochemical markers of bone metabolism. In order to additionally examine the long term effect of pinealectomy on bone mass, bone mineral density (BMD) was measured before and 3, 9, 18 and 30 months after surgery at the distal radius using peripheral quantitative computed tomography (pQCT). The full number of animals were available for all assays mentioned.

The sheep were held in large group boxes under standardized conditions and natural light, and were given access to pasture land during the day. The animals were fed twice daily with silage, hay and straw as well as water ad libidum. Their body weight was measured biweekly.

### Surgical procedures

All surgical procedures were performed under general anaesthesia with Isoflurane and intravenous Buprenorphine 0.3 mg/ml. For bilateral ovariectomy the ewes were placed in supine position, midventral laparotomy was performed, with care being taken to ligate doubly the ovarian ligament, artery and vein.

**Figure 1:**
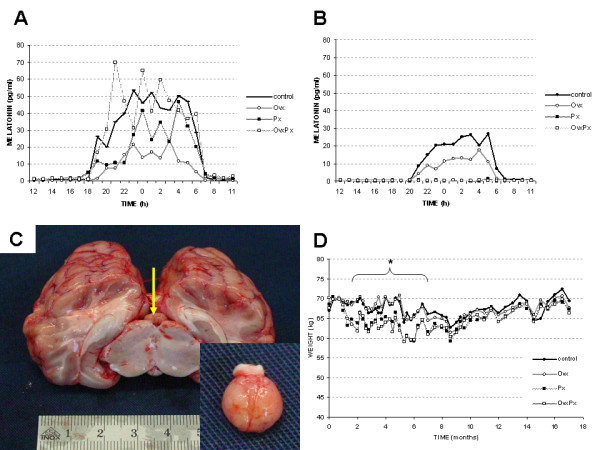
**Melatonin levels, pineal gland and body weight**. The day profiles of serum melatonin levels (mean values) before (A) and after (B) surgery demonstrate the absence of nocturnal melatonin expression after pinealectomy. Anatomy of the ovine brain (C): The pineal gland (arrow) is located on the collicular plate of the brainstem and recognized by the characteristic color and shape (C). The course of the mean body weight (D) shows lower values in the post-operative period for the Px and OvxPx group compared to control (*: p < 0.05 at some time points).

For pinealectomy a parieto-occipital approach was used as described[23,24] with the skull fixed into a stereotactic frame (Davis Kopf Instruments Inc., Tujunga, USA). A triangular craniotomy was performed, the dura mater was cut by scissor and reflected back. The left occipital lobe was carefully pushed laterally revealing the falx cerebri and its caudal extension the tentorium cerebelli. The great vein of Galen runs anterior to the pineal gland and acts as a guiding structure. The collicular plate was fully exposed, the pineal gland recognized by the characteristic color and shape (Figure 1C) and finally completely removed. Hemorrhage was meticulously controlled.

Bicortical transiliacal biopsies were taken in prone position using a trephine drill (internal diameter 6.4 mm) at 1 cm below the iliac crest. The first biopsy was randomly distributed to the right and left pelvic bone and the second biopsy was taken at the contralateral side.

### Structural parameters in micro-CT

After harvesting transiliacal biopsies and immediate fixation in 70% Ethanol the trabecular micro-structure was evaluated using a μCT imaging system. The microtomographic imaging system (μCT 40, Scanco Medical, Bassersdorf, Switzerland) was equipped with a 5 μm focal spot X-ray tube as a source. The spatial resolution was 22 μm in all directions. The volume of interest included a cube with a side length of 4 mm and was binarized using a uniform threshold of 300 (30% of the gray scale). The scans were automatically reconstructed to a 3-D cube and analyzed by associated analysis software for bone volume to tissue volume ratio (BV/TV), bone surface to bone volume ratio (BS/BV), trabecular number (Tb.N), trabecular thickness (Tb.Th), trabecular separation (Tb.Sp) and structural model index (SMI)[25].

### Histomorphometry

The biopsies were dehydrated, embedded in Polymethylmetacrylate (PMMA) and sections of 6 μm were cut in the sagital plane, then stained with Giemsa-eosin. Two pairs of slices of each biopsy with a distance >1000 μm between the pairs, were processed for histomorphometry. Pictures were obtained with a light optical microscope (Axioplan 2 imaging, Carl Zeiss, Germany) and a digital camera (Axiocam, Carl Zeiss, Germany). The static derived bone histological parameters according to the guidelines outlined by the ASBMR [26] were calculated by histomorphometric software (KS400, Version 3.0, Carl Zeiss, Germany). The outcome variables included: cortical width (Ct.Wi), bone area to total area ratio (B.Ar/T.Ar), osteoid area to bone area ratio (O.Ar/B.Ar), Osteoid perimeter to bone perimeter ratio (O.Pm/B.Pm), eroded perimeter to bone perimeter ratio (E.Pm/B.Pm), and osteoclast perimeter to bone perimeter ratio (Oc.Pm/B.Pm).

### Bone markers

Serum samples were stored at -20°C until assay. Serum bone specific alkaline phosphatase (bALP) levels were determined by ELISA, using the METRA BAP EIA kit (TECOmedical AG, Suhr, Switzerland). Control samples were within the range indicated by the manufacturer: 14.9 and 15.4 U/L for low control (range 9.8 - 21.1 U/L); 64.3 and 86.6 U/L for high control (range 62.0 - 97.2 U/L).

Deoxypyridinoline (DPD) crosslinks in sheep urine were measured as recently described using the METRA DPD ELISA (TECOmedical AG, Suhr, Switzerland). The DPD results were corrected for urinary concentration by creatinine[10].

For a quantitative measure of the excretion of pyridinoline (PYD) crosslinks in serum, the METRA Serum PYD EIA kit (TECOmedical AG, Suhr, Switzerland) was used. Control samples were measured as 2.0 and 2.3 nmol/L for low control (range 1.6. - 2.6 nmol/L) and 6.5 and 8.1 nmol/L for high control (range: 6.3 - 10.4 nmol/L).

Melatonin was measured at the Center for Experimental Pathology in Locarno by radioimmunoassay (RIA), using 125I-melatonin (DDV Diagnostica, Marburg, Germany). The sensitivity of the method was of 2 pg/ml and the interassay variability less than 15%.

### BMD-measurement

The bone mineral density (BMD in g/cm^3^) was measured by pQCT using a Densiscan 1000 (SCANCO Medical AG, Bassersdorf, Switzerland). The distal radius of both sides was examined under general anaesthesia to avoid artifacts caused by movement. After fixation of the limb in the CT scanner, the joint surface was defined on a projectional scout view as reference line and 10 consecutives slices were obtained going proximal. The effective energy of the X-ray beam was set to 40 keV and 0.5 mA. The slice thickness was 1 mm and the spatial resolution was 290 × 290 μm. A contour surrounding the radius on each cross section was detected. The cancellous bone was measured using the interior 50% of a section (μ50-CT value) and the values from both sides averaged for each animal.

### Statistical analysis

Statistical analysis was performed using SPSS software package (SPSS Inc., Chicago, USA). The normal distribution of data was not given as tested by the Shapiro Wilk test. Main effects of time within each group were tested by non-parametric analysis of variance (Friedman). Differences between treatment groups (Ovx, Px, OvxPx) and controls at each time point were analyzed by non-parametric testing (Mann-Whitney U test). The level of statistical significance was set at *P *< 0.05. Data are given in mean ± standard deviation unless otherwise stated.

## Results

All animals tolerated the surgical procedures well without any side effects. One ovariectomized sheep died at 16 months of observation due to oesophageal infection which was not related to the treatment methods of this study. The pinealectomy did not interfere with the general health of the animals. Specifically, the circadian rhythm observed by the animal care takers was in synchrony with normal ewe in the same group. Complete pinealectomy was confirmed by absence of nocturnal melatonin secretion (Figure 1A+B). During the entire observation period, the mean body weight fluctuated over time (Figure 1D). A lower body weight in pinealectomized animals was observed during the initial post-operative phase. At several time points between 2 and 6 months after surgery the differences between Px and OvxPx versus control group reached the level of significance.

**Figure 2:**
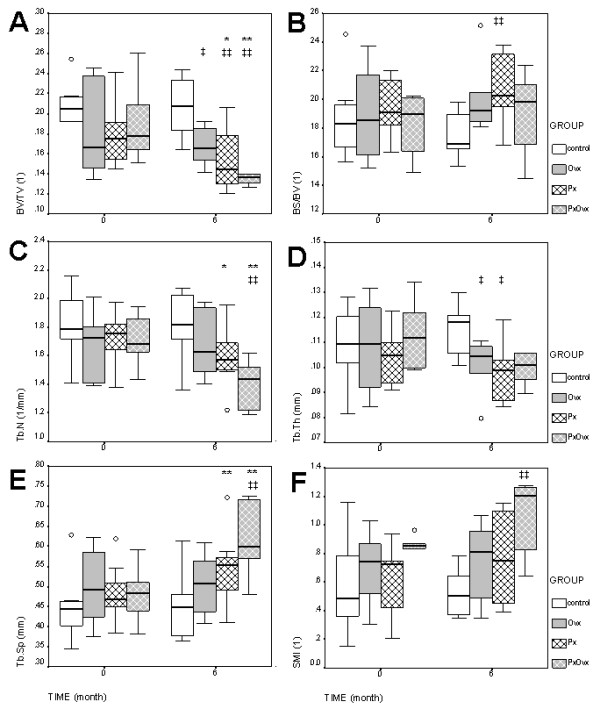
**Micro-CT-Analysis**. Morphometric analysis of trabecular bone using micro-CT scanning and 3D-reconstruction. Biopsies were taken from the iliac crest before (0 months) and after (6 months) surgery. A: bone volume (BV/TV), B: bone surface (BS/BV), C: trabecular number (Tb.N), D: trabecular thickness (Tb.Th), E: trabecular separation (Tb.Sp), F: structural model index (SMI). Boxplots show the median, interquartile range and outliers (circle) of the variables. Differences between time points: * p < 0.05, ** p < 0.01. Differences between groups compared to control: ‡ p < 0.05, ‡‡ p < 0.01.

**Figure 3:**
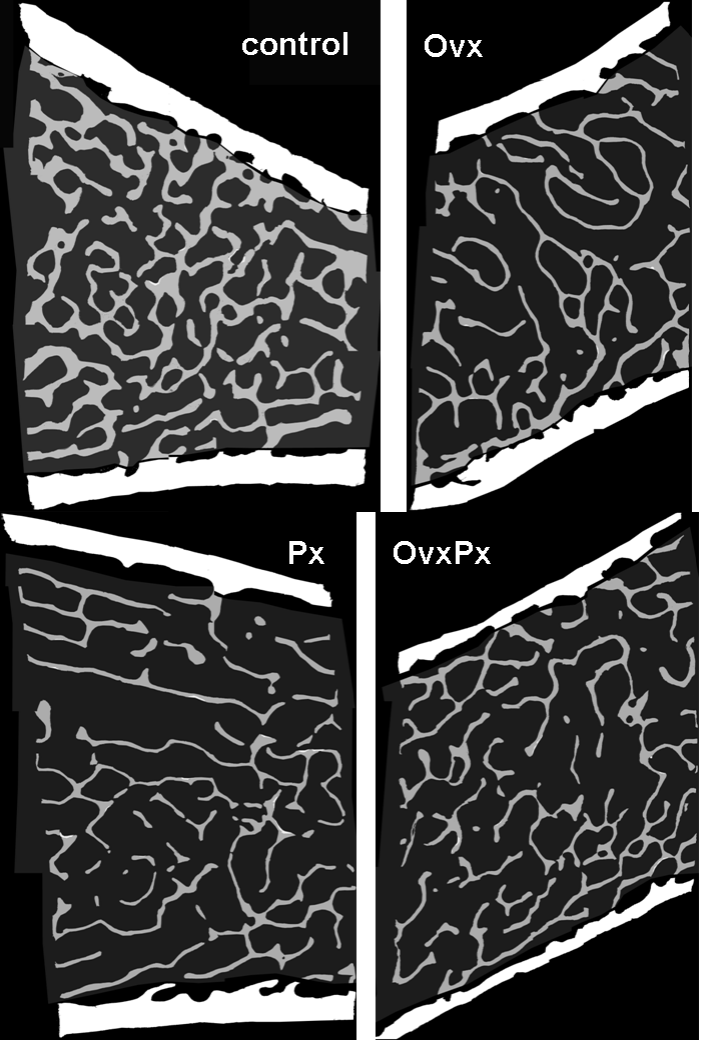
**Bone Histomorphometry**. Trabecular bone loss can be observed in all treated groups on representative slides of the iliac crest biopsies at three month after surgery. (Ovx = Ovariectomy, Px = Pinealectomy, OvxPx = Ovariectomy + Pinealectomy).

To characterize the changes in trabecular bone structure caused by pinealectomy and ovariectomy, we performed static morphometry using three dimensional micro-CT analysis of iliac crest bone biopsies (Figure 2). At the beginning of the study there were no significant differences between iliac crest biopsies of the four groups. In all pinealectomized animals (Px, OvxPx) the bone volume and trabecular number decreased after six months by -13.3% (p = 0.013) and -7.6% (p = 0.010) respectively. The trabecular separation (Tb.Sp) increased by 12.2% (p = 0.009). Some changes in the trabecular structure (Tb.N, Tb.SP) seems to be more pronounced in the OvxPx-group compared to the Px-group (Figure 2). To further characterize the bone loss caused by pinealectomy full static histomorphometry for structural parameters was performed on iliac crest biopsies(Table 1). Consistent with the data from μCT-analysis the bone area declined in all treated groups (Ovx, Px and OvxPx) (Figure 3). Ovariectomy led to a reduction in cortical thickness that was observed in the Ovx and OvxPx groups. No changes were observed on osteoid, indicating that bone formation was not disturbed. In contrast, osteoclast surface was increased in all groups treated by pinealectomy, indicating stimulation of bone resorption. The increase in eroded surface reached the level of significance in OvxPx-group.

**Table 1:** Bone Histomorphometry

	control			Ovx		
	
	0 months	6 months	*p*	0 month	6 months	*p*
Ct.Wi (μm)	858 ± 225	769 ± 358	0,249	717 ± 244	547 ± 154	0,007
B.Ar/T.Ar (%)	22,6 ± 4,3	21,7 ± 2,7	0,345	20,9 ± 5,0	18,0 ± 1,8	0,014
O.Ar/B.Ar (%)	0,225 ± 0,236	0,209 ± 0,153	0,764	0,072 ± 0,101	0,104 ± 0,116	0,314
O.Pm/B.Pm (%)	1,069 ± 1,092	0,920 ± 0,705	0,643	0,328 ± 0,481	0,504 ± 0,597	0,272
E.Pm/B.Pm (%)	1,952 ± 1,170	1,642 ± 1,036	0,293	1,625 ± 0,867	1,431 ± 0,757	0,419
Oc.Pm/B.Pm (%)	0,033 ± 0,043	0,039 ± 0,062	0,663	0,014 ± 0,033	0,027 ± 0,046	0,289
						
	**Px**			**OvxPx**		
	
	**0 months**	**6 months**	***p***	**0 month**	**6 months**	***p***

Ct.Wi (μm)	582 ± 127	637 ± 278	0,341	686 ± 125	552 ± 118	0,000
B.Ar/T.Ar (%)	19,3 ± 3,7	16,8 ± 3,7	0,015	19,7 ± 3,6	17,2 ± 2,857	0,011
O.Ar/B.Ar (%)	0,332 ± 0,323	0,327 ± 0,228	0,946	0,292 ± 0,626	0,303 ± 0,265	0,936
O.Pm/B.Pm (%)	1,545 ± 1,371	1,461 ± 1,029	0,800	1,249 ± 1,687	1,328 ± 1,065	0,846
E.Pm/B.Pm (%)	1,634 ± 1,135	1,920 ± 0,951	0,316	1,322 ± 0,862	2,230 ± 1,446	0,011
Oc.Pm/B.Pm (%)	0,012 ± 0,020	0,052 ± 0,078	0,011	0,021 ± 0,042	0,062 ± 0,068	0,017

Bone histomorphometry of biopsies from iliac crest. Six months after surgery bone area is decreased in all treated groups. Px and OvxPx increases the bone resorption indices including eroded perimeter and osteoclast perimeter indicating stimulated bone resorption.

**Figure 4:**
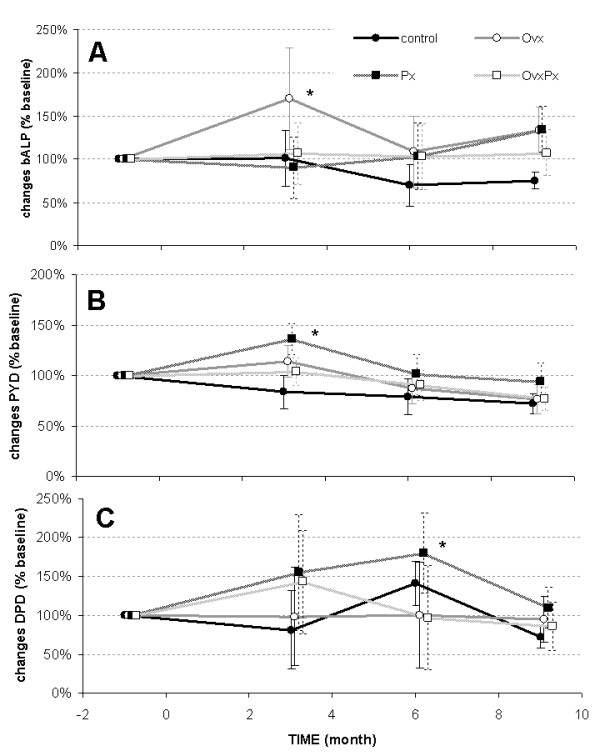
**Biochemical Marker**. Biochemical markers of bone metabolism are presented as changes relative to baseline levels. A: serum levels of bone specific alkaline phosphatase (bALP). B: Serum levels of pyridinoline (PYD). C: Urine levels of deoxypyridinoline (DPD). Differences within the group compared to base line: * p < 0.05.

The serum levels of bone alkaline phosphatase was significantly increased at 3 months after ovariectomy, but not after pinealectomy (Figure 4A). Next bone resorption markers by collagen degradation products were measured as serum pyridinoline levels and urinary deoxypyridinoline with transient increase after pinealectomy (Figure 4B+C).

**Figure 5:**
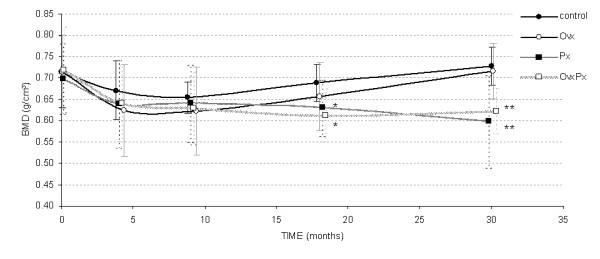
**Bone Mineral Density**. The long term course of trabecular bone mineral density at the distal radius. A rapid bone loss was observed in the initial phase after surgery with highest bone loss in Ovx sheep. At later time points a continuous bone loss was found in all pinealectomized animals in contrast to Ovx sheep, which showed a minimal increase in BMD. (*: p < 0.05, **: p < 0.01 compared to control group)

Bone mineral density was examined at the distal radius using pQCT. In order to exclude transient changes after removal of the pineal gland, the animals were followed up to 30 months and BMD was determined (Figure 5). Following surgery at the beginning of the study a tendency of decline in the cancellous bone mineral density (BMD) was observed in all groups. At later time points the BMD in control sheep slightly increased but overall remained constant at baseline levels of ~ 0.7 g/cm^3^. In ovariectomized sheep the initial cancellous bone loss of -12.7% at 3 months reached the level of significance (p = 0.008) followed by a phase of progressive increase in bone mass until 30 months post surgery. Whereas the pinealectomized animals demonstrated a continuous bone loss of -14.4% (Px, p = 0.009 between 0 and 30 months) and -14.7% (OvxPx, p = 0.001 between 0 and 30 months) until the end of the observation period (Figure 5).

## Discussion

The clinical aspects of osteoporosis are of great interest to orthopaedic surgeons, as many problems in the treatment of osteoporosis-associated fractures seem to be related to the reduction in bone mass. There is great need of a suitable animal model for experimental studies on fracture healing and fracture treatment in osteoporotic bone, as well as of a better understanding of the pathogenesis of this affliction[4].

Osteoporosis needs to be induced in animals. Ovariectomy simulates the post-menopausal estrogen depletion and a mild bone loss was observed after Ovx in sheep[8,9]. Consistent with the literature, in the present study ovariectomy leads to an initial bone loss of 12%. Interestingly, this bone loss seems to be transient and a rebound was observed after 6 months. Further studies are needed to explain this phenomenon, which may be related to the production of estrogen by alternative tissues. However, this mild reduction of BMD does not match the WHO criteria for osteoporosis in humans with a decrease of BMD by -2.5 SD.

Although there is evidence that estrogen deficiency is an important contributory factor, the pathogenesis of post-menopausal osteoporosis is multifactorial. Clinical studies suggest that characteristic changes of bone mass in postmenopausal osteoporosis, resulting from predominance of resorptive process over those leading to bone tissue formation, may be related to melatonin[16]. A few studies, particularly experimental, suggest that the main neuro-hormone of pineal gland, melatonin, can influence bone tissue metabolism[11]. Ostrowska et al have been shown that pinealectomy in rat increases collagen degradation marker as biochemical markers of bone resorption. These changes were more pronounced in ovariectomized rats[22]. Interestingly the surgical pinealectomy in newly-hatched chicks has a significant effect on the development of intervertebral disc degeneration[27].

In order to establish a large animal model for osteoporosis, we transferred the findings mentioned above to an ovine model. Bone structural parameters were determined from iliac crest biopsies indicating a significant bone loss after pinealectomy that could be attributed to an increase in trabecular separation and decrease in trabecular thickness. Some of the changes on bone structural parameters after pinealectomy were more pronounced in sheep that also underwent ovariectomy. Similar findings were reported in rat, where pinealectomy had an inducing effect upon the level of investigated markers of bone metabolism in combination with ovariectomy[22].

The changes in ovariectomized ewes observed in the present work confirmed those in previous studies. Chavassieux reported a transient increase in biochemical markers 3 months after ovariectomy and changes in cortical bone histomorphometric parameters without alterations in cancellous bone[9]. In contrast we have shown that pinealectomy in ewe decreases the cancellous bone volume without affecting the cortical structures. A synergistic effect seems to exist when Ovx and Px are combined in the present study. The bone loss affects the cancellous as well as cortical structures. Taken together, these data show that pinealectomy in ewes results in an increase in bone resorption as indicated by the histomorphometric and biochemical changes. These changes result in osteopenia observed at the iliac crest and the distal radius. These data show that Ovx and Px affects bone metabolism resulting in bone loss, supporting the assumption that the pathogenesis of post-menopausal osteoporosis is multifactorial.

At first glance this pinealectomy model including the neurosurgical approach might be a bit extreme for a widely used animal model but it seems to simulate post-menopausal osteoporosis. Leptin is known for the central regulation of bone mass and we could recently demonstrate, that the intra-cerebral injection of leptin causes bone loss in sheep[28]. However, leptin acts through the inhibition of bone formation and this is not the major cause of bone loss in post-menopausal osteoporosis. Furthermore, and in contrast to recently published sheep models for osteoporosis using leptin or glucocorticoids[28-30], there is minimal concern on animal welfare[31] and animals do not require special care after removal of the pineal gland.

Because this is a study with a limited number of animals, there are still several aspects of pinealectomy on bone remodelling that are not specifically addressed in the present work. These include the potential of melatonin application to reverse the effect of pinealectomy and to inhibit the observed bone loss, as previously shown in small animal models[15,20]. Furthermore, a significant bone loss at the iliac crest was seen at 6 months after Px and OvxPx. Although these results go along with the suspected role of melatonin on bone metabolism, the effect of pinealectomy on bone can not be distinguished from the surgical procedure only. The lack of a sham operated group is the major criticism of this study. We omitted the sham group to reduce the number of animals as required by the local ethical committee. However, in order to discriminate any effects due to the neurosurgical procedure from the influence of pinealectomy on bone, we decided to analyze the long term effect of pinealectomy. One year after surgery the effect seen on bone mass must be due to pinealectomy only and additional effects of the procedure can be excluded. Therefore bone mineral density was determined up to 2.5 years after surgery using the pQCT method. Additional bone biopsies were not taken since the healing of the former biopsy holes would have altered the newly formed bone tissue, making the interpretation of the results difficult. This long-term observation clearly shows that the influence of pinealectomy on bone is not transient and, in contrast to ovariectomy, a continuous decrease in BMD was observed. During this study extension an increase of BMD at the distal radius was observed in untreated controls. This effect might be related to an increased access to the paddock which is often associated with higher physical activity.

## Conclusions

In this experimental study pinealectomy in sheep resulted in a reduction in BMD and bone structure associated with increased bone resorption. Taken together these data provide a direct evidence for the existence of a pineal gland action on bone remodeling beyond rodents. As a consequence of this study it seems that melatonin deficiency may be a cofactor in inducing bone mass changes. Melatonin is just one candidate for hormonal modulation of osteoblast and osteoclast function and therefore this project contributes to a better understanding of bone biology and bone remodeling. Although a significant bone loss was observed in the present study, an adequate bone loss according to the definition of osteoporosis (-2.5 SD) could not be achieved. Future studies are needed to combine the numerous candidates that are able to modulate bone metabolism like melatonin, leptin and estrogen in order to obtain a synergistic effect with bone loss that simulates the situation in human.

## Competing interests

The study was fully supported by the AO Research Institute which is partly funded by royalties from licenses granted to Synthes Inc. The authors receive nothing of value and have no conflict of interest.

## Authors' contributions

ME led the project, performed the surgical procedures and CT-measurements. He drafted the manuscript. CG carried out the histology, histomorphometry and analysis of markers of bone metabolism. AB performed all neurosurgical procedures. ES participated in the design of the study, performed the statistical analysis and helped to draft the manuscript. MA conceived of the study, and participated in its design and coordination and helped to draft the manuscript. All authors read and approved the final manuscript.

## Pre-publication history

The pre-publication history for this paper can be accessed here:

http://www.biomedcentral.com/1471-2474/12/271/prepub
